# Genome-wide association studies reveal genetic variants associated with antineoplastic monoterpenoid indole alkaloid accumulation in *Catharanthus roseus*

**DOI:** 10.3389/fpls.2026.1828182

**Published:** 2026-08-20

**Authors:** Vyoma Mistry, Seongmin Hong, Chetan Kaur, Gibum Yi, Budhi Sagar Tiwari, Geung-Joo Lee, Abhishek Sharma

**Affiliations:** 1 C. G. Bhakta Institute of Biotechnology, Uka Tarsadia University, Surat, India; 2 Department of Horticulture, College of Agriculture and Life Sciences, Chungnam National University, Daejeon, Republic of Korea; 3 Department of Bio-Environmental Chemistry, College of Agriculture and Life Sciences, Chungnam National University, Daejeon, Republic of Korea; 4 School of Biotechnology and Bioengineering, Institute of Advanced Research, Gandhinagar, India; 5 Department of Smart Agriculture Systems, Chungnam National University, Daejeon, Republic of Korea

**Keywords:** anticancer, *Catharanthus roseus*, genome-wide association study, genotyping-by-sequencing, monoterpenoid indole alkaloids (MIA), vinblastine

## Abstract

*Catharanthus roseus* produces pharmacologically important monoterpenoid indole alkaloids (MIAs), yet their natural accumulation is low, limiting therapeutic exploitation. To dissect the genetic basis of natural variation in MIA accumulation, we integrated phenotypic, chemotypic, and genomic analyses of 93 *C. roseus* accessions sampled from six locations across India, including New Delhi, Lucknow, Jodhpur, Bangalore, and two locations in Gujarat: Navsari and Bardoli. Morphological characterization showed limited differentiation among locations, whereas accessions from Gujarat tended to be taller compared to other locations and more frequently white-flowered. Quantitative HPLC profiling revealed substantial accession- and location-dependent variation in total indole alkaloid levels, with Gujarat accessions showing the highest accumulation, largely driven by vindoline and catharanthine. Genotyping-by-sequencing generated 10,801 high-quality variants comprising 10,087 SNPs and 714 InDels corresponding to an average density of 19.34 variants per Mbp of the genome, revealing three genetic subgroups with overall admixed ancestry and weak geographic stratification. Genome-wide association study (GWAS) using five benchmark models identified 47 variants potentially associated with catharanthine, vindoline, and vinblastine content. These putative candidate loci were located near genes implicated in hormone signaling, mitochondrial function, nitrogen metabolism, and RNA processing, suggesting complex regulatory control of MIA biosynthesis. Notably, two missense variants in a carboxylesterase-like gene were associated with vindoline accumulation, and highly significant intergenic SNP clusters suggested putative regulatory hotspots for vinblastine biosynthesis. These results provide GWAS-based insights into the genetic architecture of MIA metabolism in *C. roseus* and nominate candidate variants for precision breeding and metabolic engineering to enhance pharmaceutical alkaloid production.

## Introduction

*Catharanthus roseus* (Madagascar periwinkle) is an evergreen medicinal plant that produces diverse secondary metabolites, including alkaloids, terpenoids, phenolics, and flavonoids, many of which have pharmacological activities (Mistry et al., 2022). Two of the alkaloids extracted from *C. roseus*, vinblastine and vincristine, have known uses as chemotherapy regimens for treating various cancers (Taher et al., 2019). These alkaloids exhibit cytotoxic effects by disrupting microtubule assembly and inhibiting mitosis. Furthermore, extracts from different parts of the plant exhibit hypoglycemic effects, suggesting potential in managing diabetes mellitus. The plant’s extracts also demonstrate hypotensive properties attributed to alkaloids and other bioactive constituents. *C. roseus* extracts have shown anti-inflammatory and antioxidant activities, making them promising candidates for alleviating inflammatory conditions and combating oxidative stress-related pathologies (Hira et al., 2024). Despite the burgeoning interest in *C. roseus* and its pharmacological attributes, several challenges persist in harnessing their full therapeutic potential, including elucidating molecular mechanisms, optimizing extraction techniques, and addressing sustainability concerns.

The biosynthesis of alkaloids in *C. roseus* primarily occurs through the monoterpenoid indole alkaloid (MIA) pathway (Thamm et al., 2016). This pathway involves a series of enzymatic reactions that convert tryptophan to various alkaloids, including vinblastine, vincristine, ajmalicine, and serpentine (Mistry et al., 2022). Key enzymes involved in this pathway include strictosidine synthase (STR), strictosidine β-glucosidase (SGD), and various cytochrome P450 enzymes (Mistry et al., 2025b). Efforts to engineer the MIA pathway using biosynthetic genes and transcription factors have been made to produce transgenic plants overexpressing MIAs pathway genes (Sharma et al., 2018b; Pan et al., 2015). However, the natural accumulation of key terpenoid indole alkaloids in *C. roseus* is extremely low, limiting downstream extraction and therapeutic exploitation. Understanding and optimizing the regulatory networks controlling secondary metabolite biosynthesis is therefore essential to improve alkaloid yield.

Various studies have been published to identify the genes and transcription factors that play a key role in the MIA pathway and accumulation (Alseekh et al., 2015; Singh et al., 2022). Conventional biparental QTL mapping is constrained by limited allelic diversity and coarse mapping resolution. Transcriptome-based approaches (e.g., RNA-seq) can identify biosynthetic genes and regulators, but they are biased toward expressed coding sequences under specific conditions and do not directly link natural DNA variation to metabolite phenotypes. Due to breakthroughs in next-generation sequencing technology, metabolomics or proteomics have been widely applied to many medicinal plant species (Mistry et al., 2022), which has provided biological significance at the metabolite or protein levels, but underlying genetic causality or low-abundance proteins should be addressed. In contrast, genome-wide association studies (GWAS) leverage historical recombination and broad natural allelic diversity to map both coding and regulatory variants, directly associating genetic polymorphisms with metabolite traits and enabling the discovery of novel pathway components and regulatory loci (Saini et al., 2025).

However, more research is needed to fully understand the complexity of the gene networks in non-model plants like *C. roseus*. In order to detect common or rare variants across germplasms, it is necessary to collect genetically diverse genotypes, allowing high-resolution loci and genome-wide scan. Unlike candidate gene approaches or single omics layers, GWAS allows the discovery of novel pathway components and regulatory loci essential for fully understanding the complex polygenic control of MIA biosynthesis. Here, we combined metabolite profiling with genotyping-by-sequencing and multi-model GWAS in a panel of 93 C*. roseus* accessions representing six focal sampling locations across India. Our objective was to identify genetic variants associated with catharanthine, vindoline, and vinblastine accumulation and to prioritize candidate loci for downstream functional validation and breeding. Our study demonstrates the utility of metabolite-based GWAS in non-model medicinal plants and establishes a foundation for improving elite *C. roseus* cultivars with enhanced therapeutic alkaloid profiles by integrating phenotypic, metabolic, and genomic information.

## Materials and methods

### Plant materials, seed germination, and phenotyping

A total of 93 C*. roseus* accessions were collected from six focal sampling locations in India, including New Delhi (28.61°N, 77.20°E, humid subtropical climate Cwa), Lucknow (26.85°N, 80.95°E, Uttar Pradesh, humid subtropical climate Cwa), Jodhpur (26.24°N, 73.02°E, Rajasthan, arid hot desert climate BWh), Bangalore (12.96°N, 77.58°E, Karnataka, Tropical Savanna Climate Aw), and two locations in Gujarat (Navsari 20.95°N, 72.95°E, Tropical Savanna Aw; and Bardoli 21.13°N, 73.11°E, Tropical Savanna Aw). Seeds were harvested from each accession and grown under controlled greenhouse conditions at the C. G. Bhakta Institute of Biotechnology, Uka Tarsadia University (Bardoli, Gujarat, India). Seeds were rinsed with distilled water, surface-sterilized in 0.1% HgCl_2_ for 60 s, and rinsed at least three times with sterile distilled water. Five plants per accession were grown in individual pots placed together in series containing garden soil, followed by watering and standard horticultural practices applied uniformly across accessions to minimize environmental variance in the greenhouse. Under the standardized growth conditions used in this study, biological replicates exhibited no obvious phenotypic differences during the experimental period. Accordingly, one representative individual from each accession was selected for downstream genotyping and GWAS analysis.

Morphological traits were assessed for each accession at defined developmental stages and included plant height, leaf size and shape, and flower color. For categorical traits, including flower color and leaf shape, trait frequencies were calculated by sampling location.

### Secondary metabolite profiling

Leaves from the lower axillary buds of 8-week-old plants were used for alkaloid isolation and HPLC-mediated identification. 100 mg of oven-dried (50–60 °C) leaves were taken and extracted with HPLC-grade methanol (3 x 10 mL) at room temperature (Sharma et al., 2018a). Methanolic extracts were pooled and concentrated under vacuum, then adjusted to a defined final volume. The extract was diluted with an equal volume of distilled water and acidified with 3% HCl prior to hexane washing. The aqueous fraction was basified to pH 8.0 with ammonia and extracted with chloroform. The chloroform fraction was dried over anhydrous sodium sulfate and evaporated to dryness.

HPLC was performed on a Shimadzu LC-2030 Plus system equipped with an RP-18e reverse-phase column, specifically the Shimadzu Prominence-I LC - 2030 plus gradient & auto-inject HPLC system (Poway, California, USA) fitted with a PDA detector. The mobile phase consisted of acetonitrile and 100 mM ammonium acetate (pH 7.3) mixed at 50:50 (v/v) delivered at an isocratic flow rate of 0.5 mL/min, with an injection volume of 10μl, with a run time of 20 mins (Mistry et al., 2025a). Vindoline, catharanthine, vinblastine, and vincristine were quantified from MIAs isolated from each accession with three technical replicates by comparing their retention times and UV–Vis spectral profiles at 254 nm with those of authentic standards purchased from Sigma-Aldrich (St. Louis, Missouri, USA) using a PDA detector. The stock solution (1mg/ml) of catharanthine, vindoline, vinblastine, and vincristine was prepared in methanol, and different amounts of these were used for the preparation of the calibration curve, linear in the range of the working concentration of (0.25-25μg). The LOD and LOQ values were calculated as described by Gupta et al. (2005) for catharanthine, vindoline, vinblastine, and vincristine based on 3 and 10 times the noise level, respectively.

### DNA extraction and quality check

Genomic DNA was extracted from young leaf tissue using the Qiagen DNeasy Plant Pro Kit following the manufacturer’s protocol (Qiagen, Arlington, VA, USA). Briefly, the 50-100mg plant tissue was homogenized using 500 μl CD1 solution in a 2ml tube, followed by vigorous vortexing for 10 minutes. Subsequently, centrifugation at 12,000 x g for 2 minutes was done to separate the cellular debris, and the supernatant was collected in a 1.5 ml collection tube. 200 μl CD2 solution was added to the supernatant, followed by vortexing and subsequent centrifugation. The resulting supernatant was supplemented with Buffer APP, loaded onto an MB Spin Column, and centrifuged for DNA binding. Subsequent washing steps with Buffer AW1 and Buffer AW2 facilitated the removal of impurities, yielding purified DNA bound to the column matrix. Elution was done by adding EB buffer to the column, followed by centrifugation. DNA quality was assessed using agarose gel electrophoresis and a NanoDrop 2000 spectrophotometer (Thermo Fisher Scientific, Waltham, MA, USA) to determine the OD260/OD280 ratio. DNA was quantified using Qubit 2.0 fluorometry (Thermo Fisher Scientific, Waltham, MA, USA). Samples with an OD260/OD280 ratio within the range of 1.8 to 2.0 and total DNA >1.5 μg were used for subsequent library preparation.

### GBS library preparation and sequencing

Genomic DNA (0.3–0.6 µg per sample) was digested with an optimized restriction enzyme set ApeKI and MspI selected by in-silico digestion against the chromosome-level *C. roseus* reference genome (Xu et al., 2023), with a size selection range kept between 300 and 400bp, following Aguirre et al. (2019) recommendations. Barcoded adapters compatible with the restriction overhangs and Illumina P5/P7 sequences were ligated to digested fragments. Libraries were PCR-amplified, pooled, and size-selected for specifying target fragment range. Library quality was evaluated using Qubit concentration, Agilent 2100 Bioanalyzer insert size, and qPCR quantification. The pooled libraries with effective concentration >2 nM was used for paired-end sequencing performed on an Illumina NovaSeq 6000 platform (Illumina, San Diego, CA, USA) with a read length of 150 bp at each end (2 x 150 bp). The data generated in this study have been deposited in the NCBI BioProject database under accession number PRJNA1469536 (**Supplementary Table 1**).

### Read processing, alignment, and variant calling

Raw reads were filtered by Trimmomatic software with default parameters. Clean reads were aligned to the *C. roseus* reference genome (Xu et al., 2023) using BWA-MEM v.0.7.17 with default settings (Li and Durbin, 2009). Alignments were converted into BAM format files, sorted, and indexed using SAMtools v.1.17 (Li et al., 2009) Joint genotyping was performed using GATK v4.1.8.1 based on the GenomicsDBImport followed by HaplotypeCaller commands (Poplin et al., 2017). Variants were filtered using options of minor allele frequency (MAF) ≥ 3%, heterozygosity rate < 50%, and missing rate ≤ 40%. Subsequently, the filtered genotype dataset was imputed using KNN imputation implemented in TASSEL v5.0 software (Bradbury et al., 2007). Imputation accuracy was evaluated through 10 iterations of randomly masking genotypes at 402 variant positions that exhibited less than 5% missing rate in the population.

### Population genetics analysis

Principal component analysis (PCA) was performed on the imputed genotype dataset using TASSEL v5.0 (Bradbury et al., 2007). Subsequently, LD pruning was performed using PLINK v1.9 (Chang et al., 2015) with a window size of 50 variants, a step size of 10 variants, and an *r^2^* threshold of 0.4 to obtain a representative set of variants within linkage disequilibrium (LD) blocks for downstream analyses. Bayesian clustering analysis was performed using STRUCTURE (Pritchard et al., 2000) implemented in the StRauto pipeline (Chhatre and Emerson, 2017) based on the LD pruned genotype dataset, with K values ranging from 2 to 10, a burn-in period of 50,000, 50,000 monte carlo markov chain (MCMC) iterations, and 10 independent runs for each K value. Delta K was computed from the structureHarvester.py script with the –evanno option, and the results were visualized by ggplot2 (Wilkinson, 2011). A maximum-likelihood phylogeny was built using IQ-TREE 2 with the best-fit substitution model by ModelFinder automatic model selection and 1,000 ultrafast bootstrap replicates (Minh et al., 2020).

### Genome-wide association study

GWAS was performed in GAPIT3 using five models, including GLM, MLM, MLMM, FarmCPU, and BLINK (Wang and Zhang, 2021). To control population structure, the ancestry coefficient (Q matrix) inferred from STRUCTURE K = 3 was included as covariates in the GWAS models. For mixed-model approaches, a kinship matrix calculated from genome-wide markers was used to account for relatedness among accessions. Significant variant-trait associations were identified using the Bonferroni-corrected threshold of −log_10_(*P-value*) > 5.33 (0.05/number of markers), whereas suggestive associations were defined as −log_10_(*P-value*) > 4.03 (1/number of markers). Manhattan and QQ plots were generated using the R package qqman (Turner, 2014).

### Variant annotation and candidate-gene proximity

The associated variants were annotated using SnpEff software v.5.2 (Cingolani et al., 2012) against the *C. roseus* predicted gene models (Xu et al., 2023). To map the location of known MIA pathway genes onto the *C. roseus* reference genome assembly, BLASTP was performed using curated protein sequences reported in a previous study (Liu et al., 2007) against the predicted proteome in *C. roseus* reference genome assembly. The BLASTP results passing the similarity thresholds of Hong et al. were retained (Hong et al., 2021). The chromosomal distribution of MIA-like genes and GWAS hits was visualized using the RIdeogram R package (Hao et al., 2020).

## Results

### Phenotypic characterization and identification of indole metabolites in the *C. roseus* population

**Figure 1:**
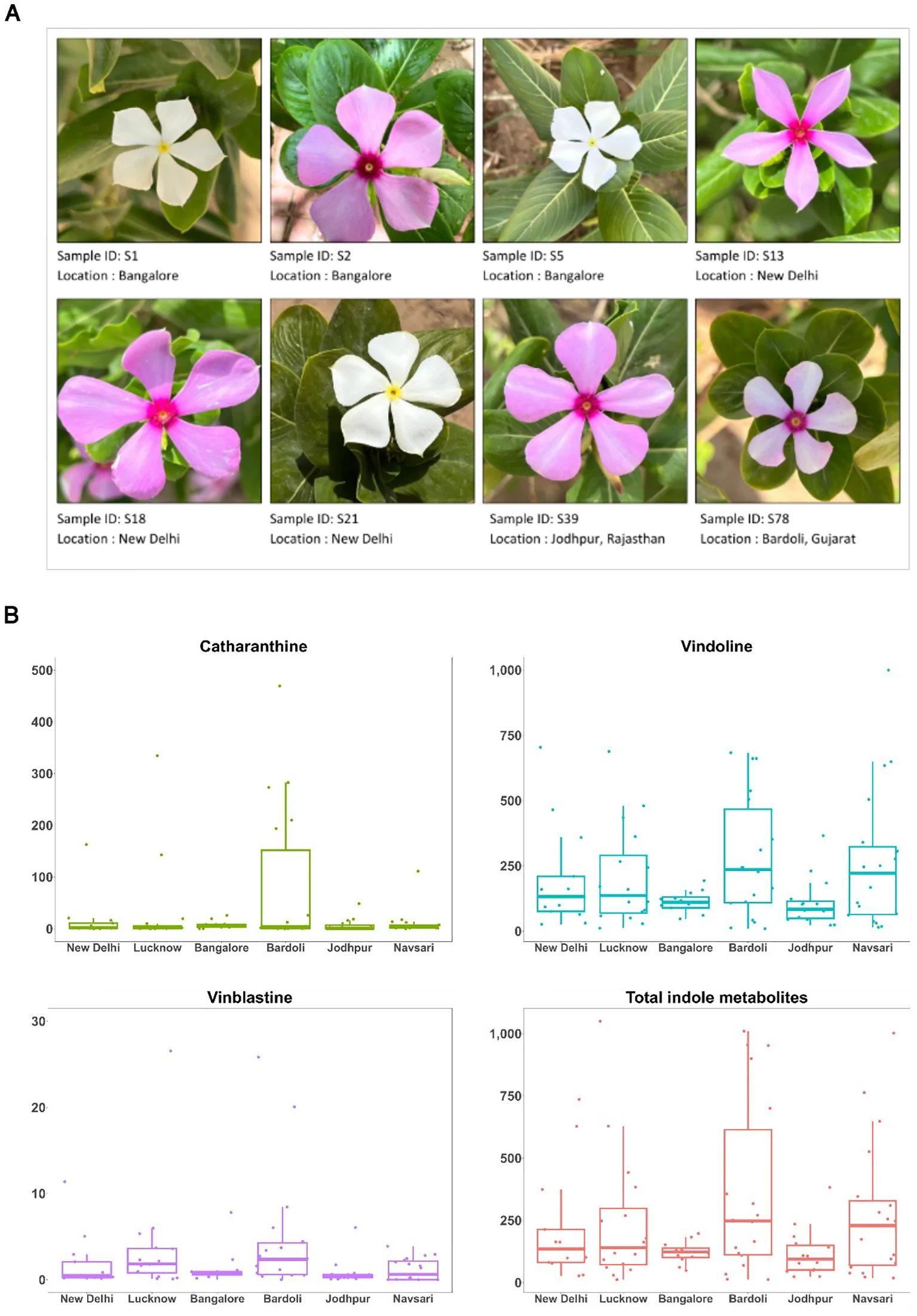
Overview of the surveyed phenotype distribution for *Catharanthus roseus* in this study. **(A)** Representative accessions of *C. roseus* collected from six locations in India **(B)** Distribution of secondary metabolites for 93 accessions.

Morphological traits and indole metabolite contents were analyzed in a *Catharanthus roseus* population comprising 93 accessions collected from six distinct geographic regions across India New Delhi, n = 13; Navsari, n = 19; Bardoli, n = 18; Lucknow, n = 16; Jodhpur, n = 15; and Bangalore, n = 12 (**Supplementary Table 1**; **Figure 1A**). From Gujarat, 19 accessions were collected from Navsari and 18 from Bardoli. Additionally, 13 accessions were collected from New Delhi, 16 from Lucknow, 15 from Jodhpur, and 12 from Bangalore. White-flowered individuals were predominant across locations (**Figure 1A**). The highest frequency of pink flowers was recorded in the Jodhpur population (40%), followed by Lucknow and New Delhi (both 31%). In contrast, Bardoli and Navsari showed lower frequencies of pink individuals (11% and 16%, respectively), with Bangalore exhibiting the lowest at 15%. Plant height differed modestly among locations, with Bardoli and Bangalore showing taller plants on average, over 76 cm. Leaf shape was predominantly oval, with a small fraction of elongated leaves in some locations, such as Bangalore, New Delhi, Jodhpur (Rajasthan), and Bardoli (Gujarat) (**Supplementary Table 1**).

The PDA-based HPLC spectral analysis enabled reliable identification of alkaloids through characteristic absorbance patterns and spectral homogeneity across chromatographic peaks. The LOD and LOQ values for catharanthine, vindoline, vinblastine, and vincristine were found to be 1.9, 1.0, 1.3, and 0.9 μg/ml and 5.9, 3.2, 4.2, and 2.9 μg/ml, respectively. HPLC profiling of catharanthine, vindoline, and vinblastine revealed substantial accession-level variation among them, with descriptive differences among sampling locations in total (sum of the three metabolites) indole alkaloid content, whereas vincristine was not detected in any accession (**Figure 1B**; **Supplementary Figure 3**). Vindoline, which is one of the precursors for the vinblastine, accounted for the largest proportion of total indole alkaloids across the panel. Accessions from Bardoli showed the highest observed mean total indole alkaloid content in this dataset (317.23 µg/ml), driven largely by catharanthine (76.64 µg/ml) and vindoline (236.14 µg/ml), followed by Navsari and Lucknow (**Supplementary Table 2**; **Figure 1B**). New Delhi (Central India) and Bangalore (Southern India) exhibited intermediate mean indole alkaloid totals, at 205.65 µg/ml and 117.27 µg/ml, respectively, reflecting moderate vindoline but low catharanthine accumulation. Notably, Jodhpur (Western India) had the lowest mean total alkaloid concentration (105.84 µg/ml), primarily due to minimal catharanthine and vinblastine levels, despite moderate vindoline content.

### Variant discovery and genome distribution

**Figure 2:**
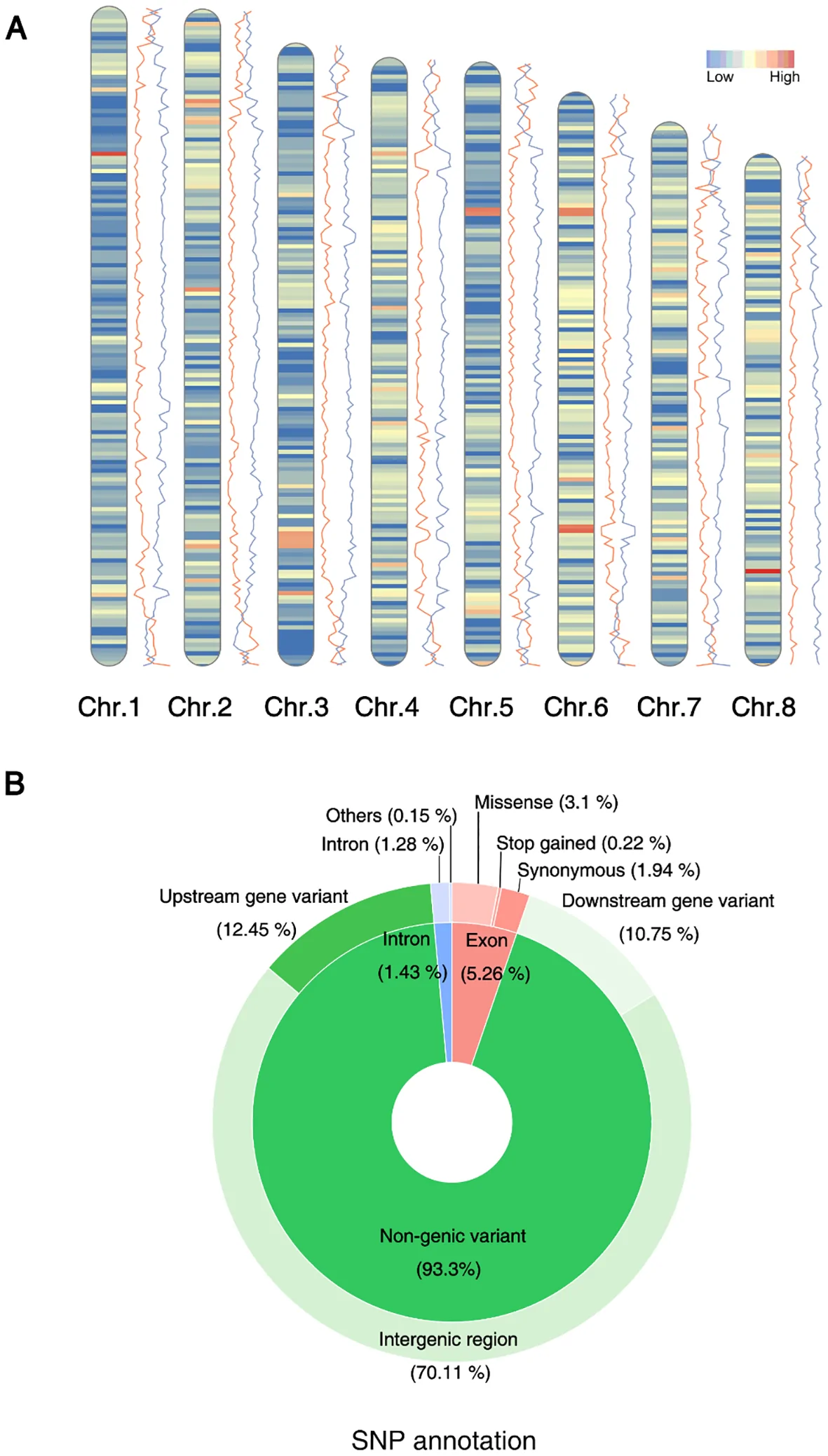
Overview of genotype data of 93 *C. roseus* accessions. **(A)** Variant density of genotype data based on the *C. roseus* reference sequence; the heatmap in the chromosome plot indicates genotype density in 100 kb sliding windows; the orange and blue lines beside chromosome plot are gene and repeat density. **(B)** The variant annotation results for SNPs in this study.

Genotyping-by-sequencing (GBS) of 93 *Catharanthus roseus* accessions generated 31.03 Gbp of sequence data, with an average of 333.65 Mbp per accession (**Supplementary Table 3**). After HaplotypeCaller-based joint variant calling and hard filtering, a total of 1,206,780 variant positions were identified across the 93 *C. roseus* accessions. To remove ambiguous variant positions for downstream analyses, filtering based on the MAF and missing rate retained 10,801 genome-wide variant positions (**Figure 2A**), which were subsequently imputed with an accuracy of 83.61 ± 0.68% (**Supplementary Table 4**). The final dataset consisted of 10,087 SNPs and 714 InDels, including 286 insertions and 428 deletions. The overall sequencing depth across the reference genome was low, averaging 0.13 X, because GBS primarily targets genomic regions adjacent to restriction enzyme sites rather than the entire genome. However, the 10,801 retained variant sites were supported by an average read depth of 4.90 X, exceeding the minimum depth threshold of three reads per identified genotype alleles (**Supplementary Table 3**). Variants were distributed across the eight *C. roseus* chromosomes, with an average density of 19.34 variants per Mbp. The highest variant density was observed on chromosome 6 (47.65 variants/Mbp), whereas the lowest density was observed on chromosome 1 (11.49 variants/Mbp). Most variants were located in intergenic regions (70.11%), whereas a smaller proportion from genic regions was located in exon (5.26%) and intron (1.43%) regions (**Figure 2B**). Functionally, the variants were categorized as synonymous, missense, or nonsense mutations, with the majority classified as intergenic and non-coding variants.

### Genetic diversity and population structure

**Figure 3:**
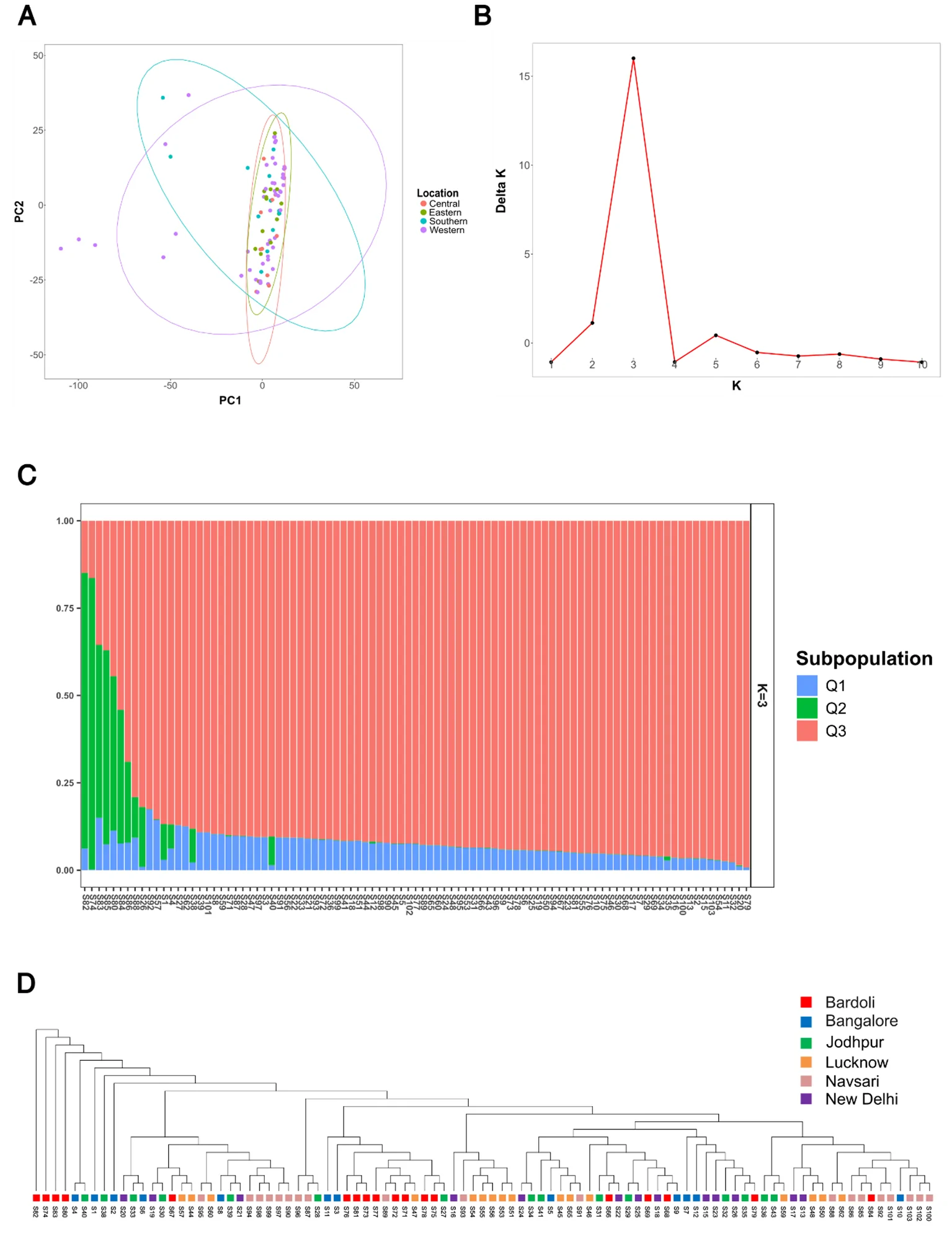
Genetic architecture of *C. roseus* population. **(A)** The PCA analysis result of genotype data. **(B)** The Delta K changes by different K values. **(C)** The STRUCTURE plot of *C. roseus* population. **(D)** The phylogenetic tree of *C. roseus* population.

The genotype PCA analysis was performed to investigate genetic variation among the 93 *C. roseus* accessions collected in India, revealing the modest population structure, with PC1 and PC2 explaining 17.23% and 8.72% of genetic variance, respectively (**Figure 3A**). Three outlier individuals (S74-1, S82-1, and S83-1) were identified, forming distinct clusters separate from the main population. Bayesian model-based clustering supported K = 3 subgroups across 93 *C. roseus* accessions (**Figure 3B**), with many accessions showing admixed ancestry within the Indian *C. roseus* accessions in this study (**Figure 3C**). A phylogenetic tree was constructed based on pairwise genetic distances among the 93 accessions (**Figure 3D**). The phylogenetic tree was broadly consistent with PCA and STRUCTURE results, and did not show strong geographic clustering, suggesting gene flow and germplasm exchange among locations. However, genome-wide linkage disequilibrium (LD) decay could not be reliably estimated due to the limited marker density and the use of imputed genotype data in this study.

### Assessment of genomic inflation in multi-model GWAS analyses

GWAS was performed using five benchmark models (GLM, MLM, MLMM, FarmCPU, and BLINK) to identify SNPs associated with the biosynthesis of the monoterpenoid indole alkaloids (MIAs) vinblastine, vindoline, and catharanthine in 93 *Catharanthus roseus* accessions. Genomic inflation factors (λGC) were compared before and after the removal of three outlier accessions (S74-1, S82-1, and S83-1) across five GWAS models with the raw phenotype dataset (**Supplementary Table 5**). The λGC values ranged from 0.51 to 1.29, with most values at or below unity, suggesting conservative association statistics. These values showed only minor changes following outlier removal, indicating that the exclusion of outlier accessions had limited effects on the λGC. Although slight model-dependent variation was observed, particularly for vindoline in the BLINK model, most λGC values remained relatively stable across metabolites and GWAS methods.

The raw metabolite phenotypes showed highly right-skewed distributions and excessive kurtosis, indicating substantial deviations from normality (**Supplementary Figure 1A**). To improve the robustness of downstream GWAS analyses, log_10_-transformation was applied to the raw phenotype data, resulting in markedly reduced skewness and kurtosis and more normalized phenotype distributions (**Supplementary Figure 1B**; **Supplementary Table 6**), therefore, the biologically representative raw phenotypes were retained for the primary analyses, with transformed-data results provided as a sensitivity analysis. Comparison of multi-model GWAS results between raw and log_10_ transformed phenotype datasets indicated that genomic inflation factors were improved after log_10_-transformation (**Supplementary Table 7**).

### Identification of associated variants by multi-model GWAS of indole metabolites

Using the content of three indole metabolites and the genotype dataset derived from 93 *C. roseus* accessions comprising 10,801 variant positions, a total of 47 trait-associated variants were identified through multi-model GWAS analyses (**Supplementary Table 8**). In the GWAS using log_10_-transformed phenotypes, a total of 4 variants (3 SNPs and 1 deletion) surpassed the suggestive association (−log_10_(1/10,801) = 4.03), with one SNP (Chr2_2158343) exceeding the genome-wide Bonferroni-corrected threshold (-log_10_(0.05/10,801) = 5.33). For the raw indole metabolite contents, a total of 46 variants (41 SNPs, 3 deletion and 2 insertion) exceeded the suggestive threshold, including 18 variants that surpassed the Bonferroni-corrected threshold. Of these, 4, 1, and 14 candidate variants exceeding the Bonferroni-corrected threshold were associated with catharanthine, vindoline, and vinblastine, respectively (**Table 1**). Notably, Chr2_2158343 was consistently detected as a candidate variant for catharanthine and vinblastine under five GWAS models in raw phenotype data, surpassing the Bonferroni-corrected threshold using the raw phenotype data. However, for the log_10_-transformed data, this variant reached the Bonferroni-corrected threshold in both BLINK and FarmCPU models for vinblastine, while it exceeded the suggestive threshold in the other three GWAS models. These results highlight a small subset of candidate loci that were reproducibly detected across phenotype treatments and GWAS models, among which Chr2_2158343 showed the most consistent association pattern in this multiple GWAS analysis.

**Table 1:** Variants associated to the raw phenotype data for three indole metabolites in *C. roseus*.

#	Variant	Type	REF	ALT	Metabolite	GWAS models				
						BLINK	FarmCPU	MLMM	MLM	GLM
1	Chr1_17228092	SNP	C	T	Catharanthine	8.00	13.06	8.88		
2	Chr1_17244708	SNP	GC	G	Vinblastine			5.45		
3	Chr1_17244733	SNP	A	T	Vinblastine	6.63		6.81		
4	Chr1_63873358	SNP	A	G	Vindoline	6.76				
5	Chr2_2158343	SNP	A	G	Catharanthine	16.89	12.44	10.5	5.53	5.61
6	Chr2_2158343	SNP	A	G	Vinblastine	22.47	9.2	12.93	8.19	8.19
7	Chr5_52290369	SNP	G	A	Catharanthine	6.39				
8	Chr6_23572093	SNP	G	A	Vinblastine			7.5		
9	Chr6_23572106	SNP	G	A	Vinblastine			7.5		
10	Chr6_23572244	SNP	C	T	Vinblastine			7.15		
11	Chr6_23572278	SNP	G	A	Vinblastine	9.21	7.77	8.45		
12	Chr6_23572280	SNP	G	A	Vinblastine			8.45		
13	Chr6_23572282	SNP	A	T	Vinblastine			8.45		
14	Chr6_23572291	Deletion	TG	T	Vinblastine			8.45		
15	Chr6_23572294	SNP	C	T	Vinblastine			7.86		
16	Chr6_23572302	SNP	T	C	Vinblastine			8.45		
17	Chr6_44794681	SNP	A	G	Catharanthine		5.44			
18	Chr6_51862653	SNP	T	C	Vinblastine			5.99		
19	Chr8_48716800	SNP	T	C	Vinblastine	5.41				

The values represent the -log_10_(*P-values*) for corresponding variants surpassing Bonferroni-corrected thresholds in the GWAS analysis.

**Figure 4:**
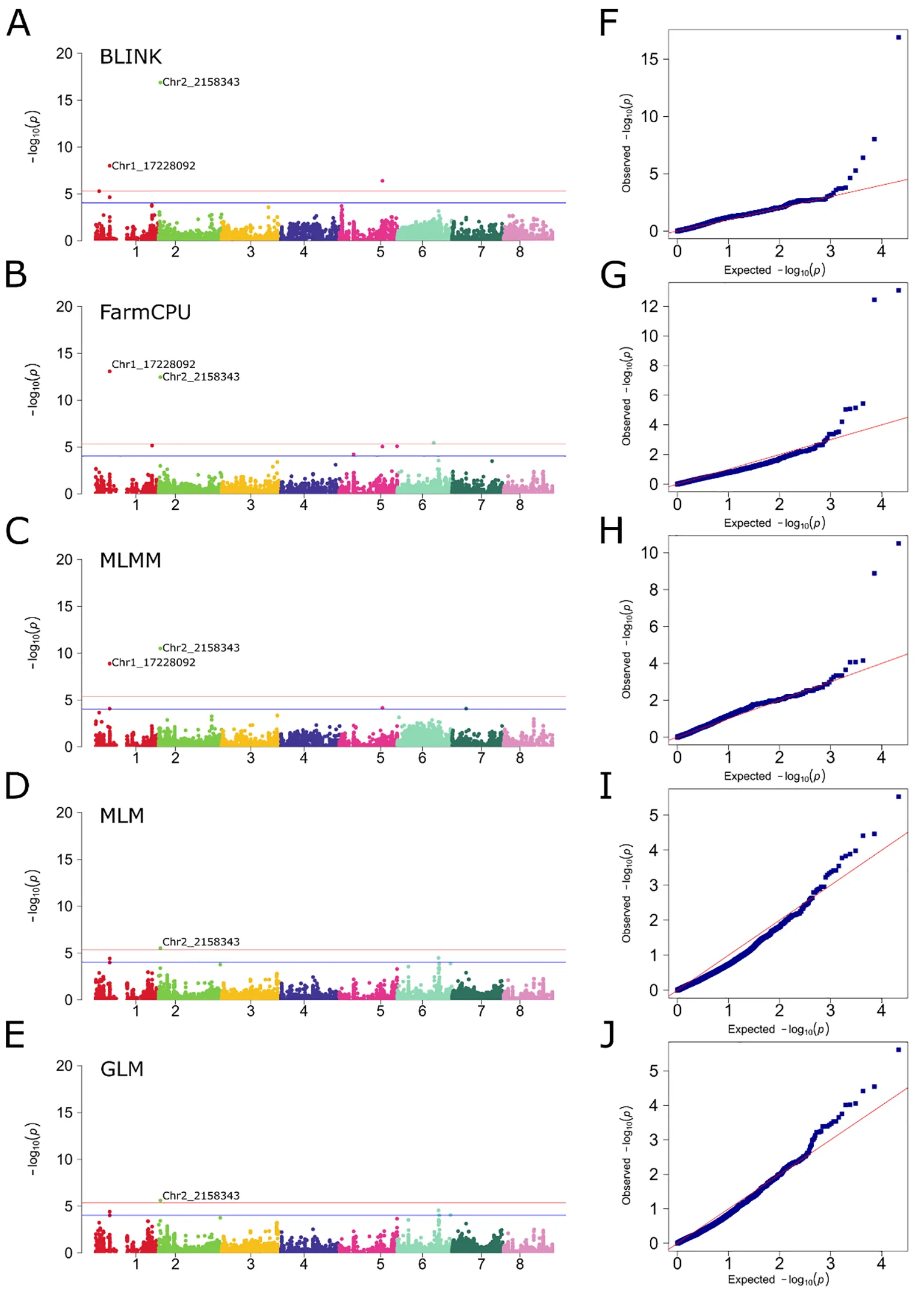
Comparison of genome-wide association results for raw catharanthine content obtained using five GWAS models. **(A–E)** Manhattan plots generated using the BLINK **(A)**, FarmCPU **(B)**, MLMM **(C)**, MLM **(D)**, and GLM **(E)** models. The horizontal blue and red lines indicate the suggestive significance threshold (−log_10_(1/10,801) = 4.03) and the Bonferroni-corrected significance threshold (-log_10_(0.05/10,801) = 5.33), respectively. Candidate loci detected above the significance threshold are labeled. **(F–J)** Quantile–quantile (QQ) plots corresponding to the BLINK **(F)**, FarmCPU **(G)**, MLMM **(H)**, MLM **(I)**, and GLM **(J)** models, showing the observed versus expected distributions of association *P-values*.

The five GWAS models showed substantial differences in the number and distribution of putative candidate variants exceeding the Bonferroni-corrected threshold (**Table 1**). MLMM detected the largest number of significant variants, whereas BLINK and FarmCPU each identified 8 and 5 variants, respectively. In contrast, GLM and MLM detected only at one variant position (Chr2_2158343), resulting in substantially fewer detected associations under the current dataset and analytical conditions. Although MLMM identified the highest number (15 variants) of significant associations, nine of these variants were concentrated within a narrow 209 bp genomic interval on chromosome 6 (Chr6:23,572,093–23,572,302), suggesting the presence of locally clustered association signals. Among the identified variants associated with indole metabolites in this study, three variants (Chr1_17228092, Chr2_2158343, and Chr6_23572278) met the Bonferroni-corrected significance threshold in at least three different GWAS models for both catharanthine and vinblastine content (**Figure 4**; **Supplementary Figure 2**). For raw vindoline content, the BLINK model identified only a single variant that exceeded the Bonferroni-corrected significance threshold, while six variants met the suggestive significance threshold (**Table 1**; **Supplementary Table 8**). Variants supported by at least three GWAS models generally exhibited higher phenotypic variance explained (PVE) values (average: 52.77, range: 0.02 ~ 95.33) than variants detected by fewer models (average: 4.84, range: 0 ~ 56.85).

### Proximity of multi-model GWAS variants to MIA pathway genes

To evaluate the potential functional impact of variants on adjacent genes, the variant annotation was conducted for all variants exceeding the suggestive threshold, using the chromosome-level reference genome assembly for *C. roseus* published by Xu et al. (**Supplementary Table 9**). Of the 47 variant positions associated with indole metabolite traits in multi-model GWAS, 44 variants were mapped to intergenic regions. The remaining three variants were located within the exon region. Among the exonic variants, Chr2_2158343 was a synonymous variant in CrChr2.175. However, two variants (Chr1_63873358 and Chr1_63873360) were identified as missense variants densely clustered within CrChr1.2583.

A total of 48 predicted *C. roseus* gene models were located adjacent to 47 variants identified on the *C. roseus genome* assembly (**Supplementary Table 10**). BLASTP analyses were conducted against Arabidopsis protein sequences in the TAIR database to annotate the putative functions of these candidate gene models. However, the BLASTP results did not reveal direct relationships with known monoterpenoid indole alkaloid (MIA) pathway genes in *C. roseus* previously summarized by Liu et al. (**Supplementary Table 11**).

**Figure 5:**
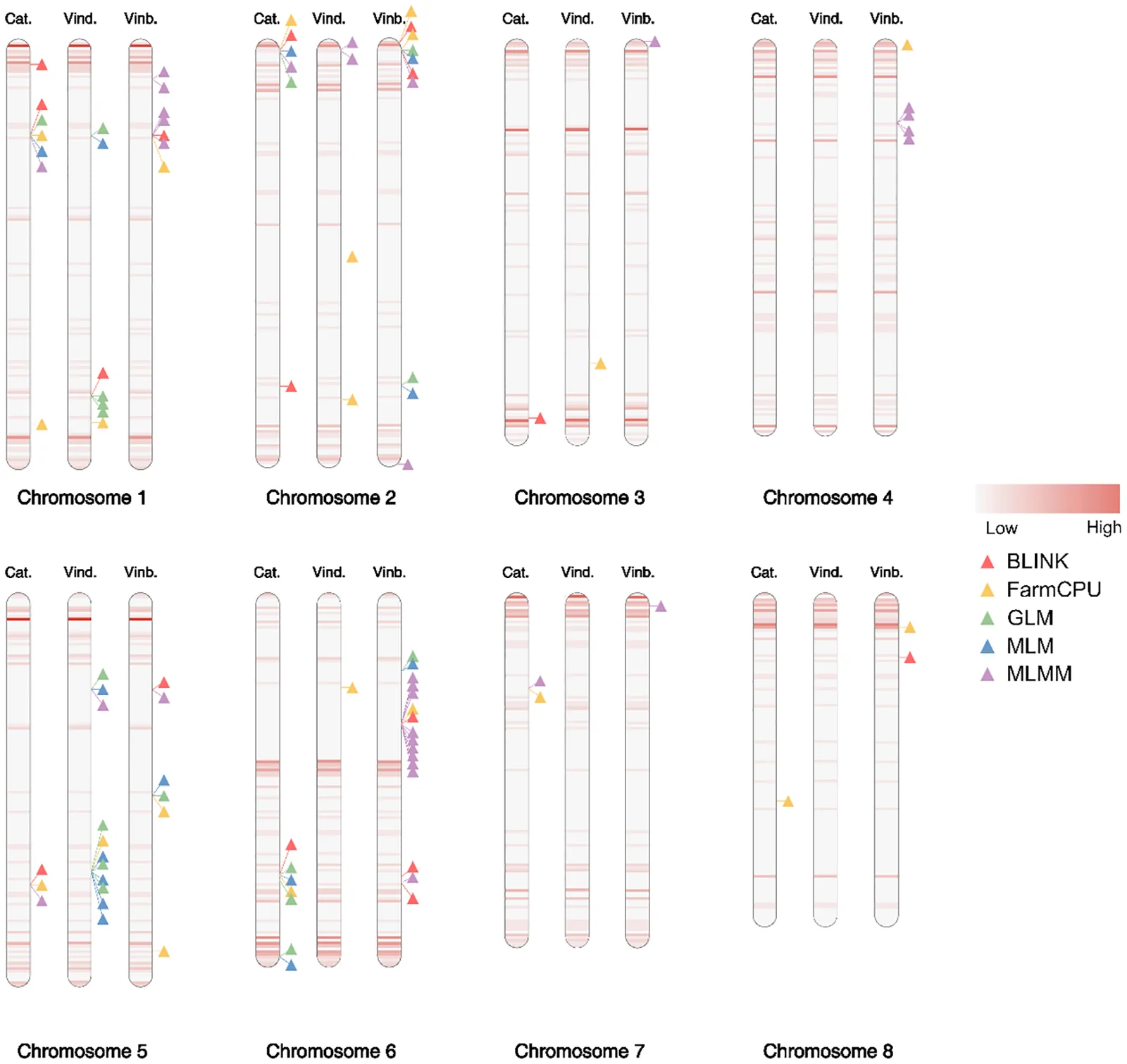
The distribution of variants potentially associated to the indole metabolite contents in this study by different GWAS models. The heatmaps in the chromosome plots indicate the number of identified indole synthesis related genes within the *C. roseus* genome assembly. The identified SNPs are colored by corresponding GWAS models.

To further explore the functional relevance of the associated variants identified by multi-model GWAS for the three indole alkaloids in this study, we confirmed whether identified candidate loci showed proximity for the genetic regions harboring gene models related to the known indole alkaloid pathway. The BLASTP analysis was performed between the protein sequences of the 23 cloned genes known to be involved in the MIAs pathway (Liu et al., 2007) and the protein sequences of the annotated all gene models from the *C. roseus* reference genome (**Supplementary Table 12**). A total of 147 gene models annotated from the *C. roseus* reference genome assembly showed high sequence similarity for 23 MIA protein sequences (**Supplementary Table 11**). Many of these MIA-like genes were in proximity to the GWAS-identified SNPs, supporting that these variants are potentially linked to regulatory elements or genes involved in indole alkaloid biosynthesis (**Figure 5**).

## Discussion

In this study, we integrated metabolite profiling and genotyping-by-sequencing to elucidate the genetic basis of monoterpenoid indole alkaloid (MIA) variation in 93 accessions of *Catharanthus roseus* (Madagascar Periwinkle) collected from six regions across India (**Figure 1**). Although the population exhibited weak geographic stratification and overall admixed ancestry (**Figure 3**), strong accession-dependent variations were observed for catharanthine, vindoline, and vinblastine, indicating substantial heritable contributions to MIA accumulation (**Figure 1B**; **Supplementary Table 2**). Geographically, accessions from Western India (Bardoli) showed the highest total indole alkaloid content, primarily driven by elevated catharanthine and vindoline levels. Accessions from both Lucknow and New Delhi exhibited moderate to high levels of indole alkaloid levels, whereas those from both Jodhpur and Bangalore showed the lower level of total alkaloid content, suggesting that these accessions contain reduced biosynthetic capacity. Interestingly, a subset of accessions with high alkaloid content was observed across different geographic origins, indicating that metabolic potential is not strictly determined by regional lineage but may be influenced by local microenvironmental selection (Katz et al., 2021) as well as underlying QTLs associated with indole alkaloid biosynthesis, as previously reported in *C. roseus* populations (Sharma et al., 2012; Chaudhary et al., 2013).

The Genotyping-by-Sequencing (GBS) approach is a cost-effective method for generating genome-wide genotype data from populations, including those of non-model species (Elshire et al., 2011). Initially, a total of 1,206,780 raw variant sites were identified through joint genotyping of 93 *C. roseus* accessions. However, a large proportion of variants was removed during filtering because high-level of missing genotypes are an inherent characteristic of GBS method, resulting from low sequencing depth and variation in restriction enzyme recognition sites among accessions (Brouard et al., 2017). Because *C. roseus* is an open-pollinated species, substantial heterozygosity is expected across the genome. However, low-depth GBS data are prone to allelic dropout, resulting in heterozygous genotypes being erroneously called as homozygous genotypes during the imputation process (Cooke et al., 2016). Despite being collected from six geographically distinct regions across India, the genotype dataset showed weak geographic clustering in the population analysis (**Figure 3**). Furthermore, dissecting the genome-wide LD structure was limited due to the sparse marker density and moderate imputation accuracy of the GBS dataset in this study. The moderate imputation accuracy observed in this study highlights a potential limitation of low-depth GBS data, where allelic dropout may lead to the underrepresentation of heterozygous genotypes in the process of genotype imputation.

Through multi-model GWAS, we identified and annotated 47 candidate variants (42 SNPs, 3 deletions, and 2 insertions) potentially associated with variation in catharanthine, vindoline, and vinblastine contents in a *C. roseus* population consisting of 93 accessions collected from India (**Supplementary Tables 8**–**10**). Two closely spaced missense variants were putatively associated with vindoline accumulation. These variants are located within CrChr1.2583, which encodes a carboxylesterase-like protein, a class implicated in the hydrolysis of hormone conjugates like methyl jasmonate (MeJA), methyl salicylate, and methyl IAA *in vitro* in other plants (Chaffin et al., 2024). Because jasmonate signaling is a key inducer of the MIA pathway, this carboxylesterase-like gene represents a putative candidate for future investigation; however, its relationship with vindoline accumulation remains speculative (Pan et al., 2018). In particular, a synonymous variant (Chr2_2158343) on CrChr2.175 was consistently identified by all GWAS models as a candidate variant associated with multiple indole alkaloids (**Figure 4**, **Table 1**, **Supplementary Figure 2**, and **Supplementary Table 8**). However, the CrChr2.175 has no known direct role in the MIA pathway and is predicted to encode SAV4, a mitochondria-localized PPR protein involved in embryo development and ethylene responses in root hair growth (Chen et al., 2022). Nevertheless, Chr2_2158343 is located near genomic regions harboring MIA pathway genes, suggesting that this region may contain candidate genes potentially involved in indole alkaloid biosynthesis in *C. roseus* (**Figure 5**).

A key limitation of the current study is that GBS captures only a subset of genomic variation. Consequently, many significant associations fall in intergenic regions are not fully resolved, limiting direct inference of causal genes connected to protein-coding genes. To address these constraints, future studies should incorporate whole-genome resequencing (WGR) or whole-exome sequencing (WES), which offer more comprehensive coverage of genic and regulatory elements. Such high-resolution approaches will be critical for pinpointing causal variants, improving functional marker development, and elucidating the genetic architecture underlying MIA biosynthesis in *C. roseus*. These efforts will ultimately accelerate the identification of key genes and regulatory networks, laying the groundwork for precision breeding and metabolic engineering strategies aimed at enhancing therapeutic alkaloid production.

Despite these limitations, the identified loci provide preliminary candidates for further validation and may eventually support marker-assisted selection or precision breeding once confirmed in independent populations. This study represents one of the few GWAS efforts in *Catharanthus roseus*, a species with a limited number of functionally characterized genes, especially within the MIAs biosynthetic pathway. Accessions from Gujarat (Navsari and Bardoli) showed high total indole alkaloid content, highlighting these populations as promising sources for breeding or metabolic engineering aimed at increasing vindoline and catharanthine, the monomeric precursors required for vinblastine biosynthesis. For translational applications, the most robust GWAS signals in the Chr6 intergenic SNP cluster should be followed by fine-mapping using whole-genome resequencing and LD-aware haplotype analysis, and by expression/allele-specific assays in relevant tissues. Also, vinblastine, a dimeric alkaloid with well-established anticancer properties, was detected in several populations, including Bardoli, Lucknow, New Delhi, and Bangalore. This result suggests that valuable chemotypes are not confined to specific geographic regions and may be exploited for targeted breeding to improve pharmaceutical yields. However, the high degree of intra-regional variation in alkaloid profiles highlights the importance of selecting elite individuals rather than relying solely on geographic origin as a proxy for desirable traits. Collectively, this work expands genetic resources for *C. roseus* and provides an exploratory GWAS framework for identifying candidate loci associated with MIA metabolism. Further validation using higher-resolution genotyping, LD-aware fine mapping, expression analysis, and functional assays will be required before these loci can be used confidently for breeding or metabolic engineering.

## Data Availability

Raw sequencing data for the 93 Catharanthus roseus accessions are available in the NCBI SRA database under BioProject accession PRJNA1469536.
