# Supplementary material for: Genome-wide association studies reveal genetic variants associated with antineoplastic monoterpenoid indole alkaloid accumulation in Catharanthus roseus
Source: Front Plant Sci. 2026 Aug 20;17:1828182. doi: 10.3389/fpls.2026.1828182 (PMC13538439; doi:10.3389/fpls.2026.1828182)
Supplement: Supplementary file 1 [file Supplementaryfile1.docx]

**Supplementary Figure 1.** Phenotypic distributions of three indole metabolites in 93 *C. roseus* accessions. (A) Raw phenotype distributions. (B) log10-transformed phenotype distributions.

**Supplementary Figure 2.** Comparison of genome-wide association results for raw vinblastine content obtained using five GWAS models. (A–E) Manhattan plots generated using the BLINK (A), FarmCPU (B), MLMM (C), MLM (D), and GLM (E) models. The horizontal blue and red lines indicate the suggestive significance threshold (−log10(1/10,801) = 4.03) and the Bonferroni-corrected significance threshold (-log10(0.05/10,801) = 5.33), respectively. Candidate loci detected above the significance threshold are labeled. (F–J) Quantile–quantile (QQ) plots corresponding to the BLINK (F), FarmCPU (G), MLMM (H), MLM (I), and GLM (J) models, showing the observed versus expected distributions of association *P-values*.

**Supplementary Figure 3.** HPLC Chromatogram of Accession A) Cr7 and B) Cr9.
